# Novel regulatory role of non-coding RNAs in ankylosing spondylitis

**DOI:** 10.3389/fimmu.2023.1131355

**Published:** 2023-02-24

**Authors:** Yanyan Fang, Jian Liu

**Affiliations:** ^1^ The First Affiliated Hospital, Anhui University of Chinese Medicine, Hefei, China; ^2^ Key Laboratory of Xin'an Medicine of the Ministry of Education, Anhui University of Chinese Medicine, Hefei, China; ^3^ Anhui Province Key Laboratory of Modern Chinese Medicine Department of Internal Medicine Application Foundation Research and Development, Hefei, China; ^4^ Institute of Rheumatology, Anhui Academy of Chinese Medicine, Hefei, China

**Keywords:** lncRNAs, circRNAs, miRNAs, ceRNA, ankylosing spondylitis

## Abstract

Ankylosing spondylitis (AS) is a type of arthritis that primarily affects the spine and involves disorders of the immune and skeletal systems. However, the exact pathogenesis of AS is not fully understood. Non-coding RNAs (ncRNAs), particularly, long non-coding RNAs (lncRNAs), circular RNAs (circRNAs), and micro RNAs (miRNAs) and their interactions have been shown to influence many biological processes such as inflammatory responses, osteogenic differentiation and apoptosis, pyroptosis, and proliferation. In addition, ncRNAs reflect the disease activity of AS. In this review, we discuss the regulatory roles of ncRNAs in AS cell functions (inflammatory responses, cellular osteogenic differentiation and apoptosis, pyroptosis, and proliferation) and their potential applications in AS diagnosis and treatment. Understanding the role of ncRNAs in the pathogenesis of AS will lay the foundation for exploring potential new therapeutic approaches for AS.

## 1 Introduction

Ankylosing spondylitis (AS) is a chronic autoimmune disease characterized by systemic inflammation and osteogenesis (1, 2), and is a highly disabling and destructive type of arthritis (3). AS affects 0.09%-0.3% of the global population (4) and occurs mostly in young people (5). It causes severe back pain and stiffness, which leads to decreased function and eventually spinal and pelvic fusion, imposing a significant burden on patients and the society (6, 7). In recent years, multiple factors contributing to the development of AS have been identified, including infections, environmental triggers, and genetic susceptibility, especially immune disorders (8–10).

Inflammation and stiffness are the primary manifestations of AS (11). Treatment with non-steroidal anti-inflammatory drugs (NSAIDs) and biologics, including tumor necrosis factor inhibitors (TNFi), interleukin-17 inhibitors (IL-17i), and more recently, Janus kinase inhibitors, has led to significant improvements in clinical symptoms and quality of life (12–14). In addition, recent studies have shown that biological agents have the potential to inhibit new bone formation in a sustained manner (11). However, for patients with a high disease activity or in whom the process of bone remodeling has already started, inhibition of inflammation will not be sufficient. Surgical intervention is inevitable to improve pain and joint motion (15). Despite recent advances in AS drug development, the functional outcome for many AS patients remains unsatisfactory. In particular, the development of new therapies is challenged by the fact that the pathogenesis of AS is still unclear (16). Therefore, new molecular targets must be identified.

Non-coding RNAs (ncRNAs) have recently attracted considerable attention caused by their critical role in biology (17). They are divided into housekeeping ncRNAs (such as transfer RNA (tRNA) and ribosomal RNA (rRNA)) and regulatory ncRNAs. Regulatory ncRNAs include micro RNAs (miRNAs), long non-coding RNAs (lncRNAs), and circular RNAs (circRNAs) (18). The competing endogenous RNA (ceRNA) hypothesis, proposed in 2011 based on experimental and theoretical studies, states that RNAs can communicate with each other and post-transcriptionally regulate gene expression by binding to miRNA binding sites (19). Different RNAs will form multiple regulatory relationships, which will eventually form a ceRNA network. Currently, studies are being conducted on the pathogenesis of ceRNA-related diseases, and various ncRNAs (e.g., lncRNAs, miRNAs, circRNAs) and ceRNA networks constructed from differentially expressed ncRNAs have been shown to be related to the pathogenesis of various autoimmune diseases (20–23), further illustrating the interconnection between different RNA molecules and the regulation of gene expression. The construction of ceRNA regulatory networks is of great importance to further understand the functions of ncRNAs. The interconnection and altered regulatory expression of different RNA molecules may also be involved in the pathogenesis of AS.

**Figure 1:**
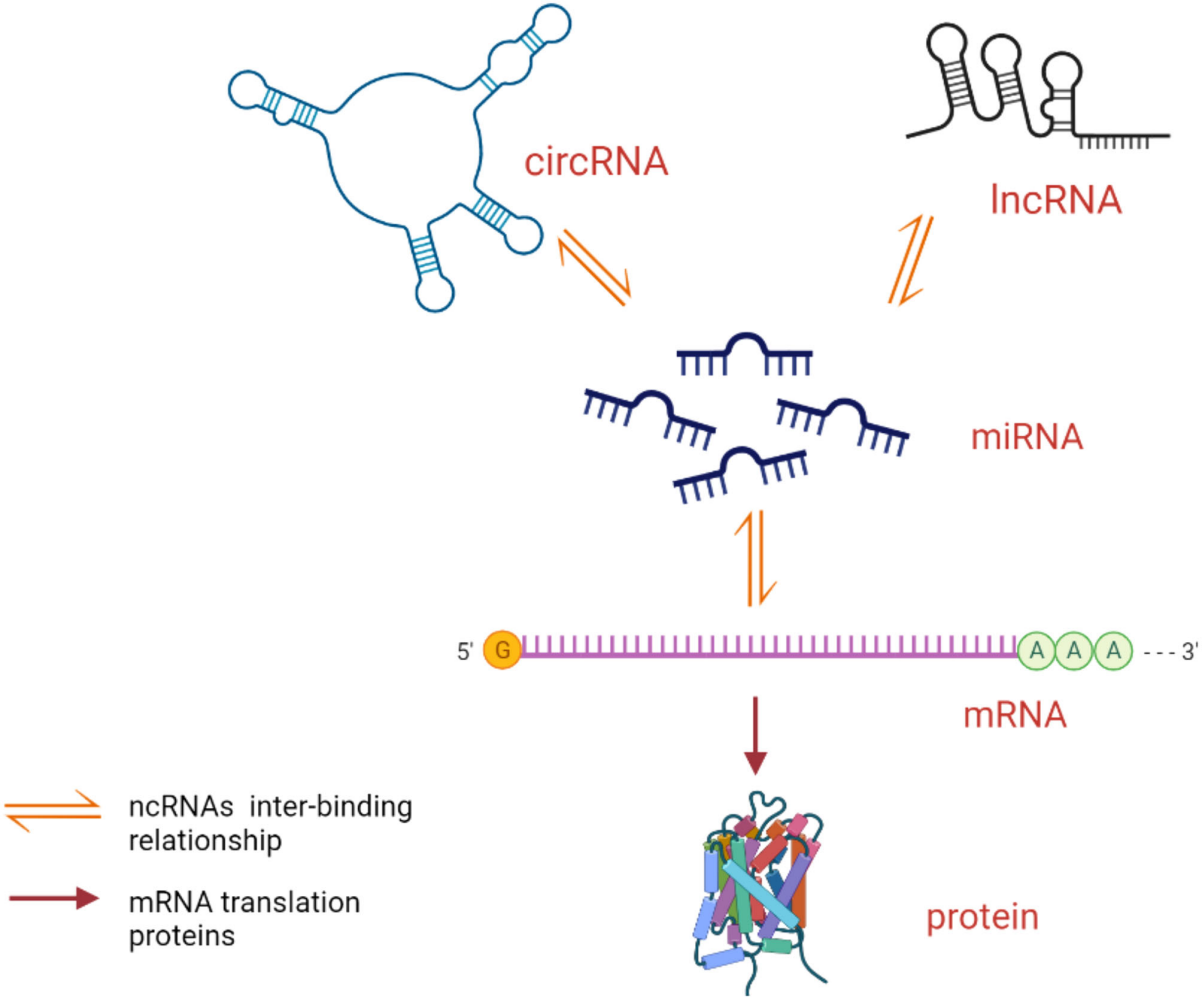
Mode of action of ceRNA network. lncRNAs/circRNAs competitively bind miRNAs to form ceRNAs to play the role of translating proteins.

In this review, we focus on the roles and possible mechanisms of three ncRNAs (lncRNAs, miRNAs, and circRNAs) in AS (**Table 1**) and discuss the role of ceRNA networks associated with lncRNAs/circRNAs in the pathogenesis of AS (**Figure 1**). This will help in the diagnosis and treatment of AS.

**Table 1:** Regulatory functions of ncRNAs in ankylosing spondylitis cells.

ncRNAs	Name	Expression	Target genes	Source	Action	References
miRNA	miR-451	down	MIF	PBMCs	Inhibits inflammation	(24)
	miR−150−5p	down	VDR	Ligament fibroblasts	Inhibits osteogenic differentiation	(25)
	miR−204−5p	down	Notch2	Ligament fibroblasts	Inhibits osteogenic differentiation	(26)
	miR−148a−3p	up	DKK1	Ligament fibroblasts	Promotes osteogenic differentiation	(27)
	miR-96	up	SOST	Osteoblasts isolated from AS mice	Promotes osteogenic differentiation and bone formation	(28)
	miR-214	up		Osteoblasts isolated from miR-214^fl/fl^ mice	Inhibits bone formation	(29)
	miR-130a-3p	down	HOXB1	T cells	Inhibits proliferation and induces apoptosis	(30)
	miR-204	down	GSDMD	FLSs	Induces pyroptosis	(31)
lncRNAs	LOC645166	down		T cells	Inhibits the activation of NF-kB	(32)
	MEG3	down	miR-146a	FLSs	Inhibits inflammation	(33)
circRNA	circ_0003307	up		FLSs	Promotes the activation of the PI3K/AKT pathway	(34)
	circ_0070562	up	TGF-β	Bone marrow-derived mesenchymal stem cells	Promotes osteogenic differentiation	(35)
lncRNA-related ceRNA	lncRNA H19-miR22-5p/miR675-5p	up	VDR	PBMCs	Promotes inflammation	(36)
	lncRNA MALAT1-miR-558	up	GSDMD	Chondrocytes	Inhibits proliferation and induces apoptosis and pyroptosis	(37)
circRNA-related ceRNA	circ_0000652- miR-1179	up	OX40L	PBMCs	Promotes inflammation	(38)
	circ_0018168-miR-330-3p	down	DKK1	Fibroblasts	Inhibits osteogenic differentiation	(39)
	circ_0056558-miR-1290	up	CDK6	Fibroblasts	Inhibits proliferation and induces apoptosis	(40)

MIF, Macrophage migration inhibitory factor; PBMCs, Peripheral blood mononuclear cells; VDR, vitamin D receptor; DKK1, Dickkopf homologue 1; SOST, sclerostin; GSDMD, Gasdermin D; FLSs, Fibroblast-like synoviocytes; MEG3, lncRNA maternally expressed gene 3; TGF-β, transforming growth factor β; CDK6, Cyclin-dependent kinase 6.

## 2 Role of ncRNAs

ncRNAs are widely present in cells and are involved in many physiological and pathological processes by regulating gene expression, cell cycle, chromatin remodeling, and epigenetic modifications (41, 42). Although ncRNAs lack the ability to encode proteins, they not only play important physiological regulatory roles in various cellular activities, but their aberrant expression and regulation are also important links in the pathogenesis of many diseases. ncRNAs are usually expressed in response to external signals, during differentiation or at specific stages of development. Their differential expression can regulate the transcription or translation of other genes or can directly interfere with signaling pathways (43).

### 2.1 miRNAs

miRNAs are endogenous ncRNAs (approximately 22 nucleotides long) that are widely found in eukaryotic organisms. They can regulate gene expression by directly targeting the promoters of bound genes or by binding to the non-protein translation region (3’-UTR) of the target messenger RNA (mRNA) to induce degradation of the target mRNA or translational repression, thereby achieving transcriptional or post-transcriptional level regulation of genes (44).

### 2.2 lncRNAs

lncRNAs are a class of ncRNAs longer than 200 nucleotides; depending on their intracellular localization, lncRNAs can play different roles (45). Cytoplasmic lncRNAs mainly play ceRNA roles by regulating the degradation or translation of target mRNAs or competitively binding miRNAs to regulate gene expression at the post-transcriptional level; cytosolic lncRNAs mainly play regulatory roles by controlling the epigenetic state of specific genes, directly participating in transcriptional regulation and variable splicing or constituting nuclear structural domains (46, 47).

### 2.3 circRNAs

circRNAs are a class of covalently closed loops without a 5’ cap and 3’ poly A tail, unlike traditional linear RNAs. circRNAs are present in high abundance and have high diversity, spatio-temporal specific expression, and highly conserved sequences; in particular, the covalent closed-loop structure protects them from nucleic acid exonuclease shearing. They have higher stability than linear RNAs (48). The molecular biological functions of circRNAs in genetic or epigenetic regulation are becoming clear, including competitive binding of miRNA, regulation of transcription and variable splicing, and interaction with RNA-binding proteins (49).

## 3 Regulatory functions of ncRNAs in AS cells

### 3.1 AS cell inflammatory responses

Immune-mediated inflammatory response is a key aspect of AS, with the IL-23 and TNF pathways being the main effector pathways (50). The inflammatory response in AS is reflected by the dysregulation of inflammatory cytokines in blood and tissues *in vivo*. High expression of cytokines activates inflammatory signaling pathways, causing an inflammatory response in the body leading to AS-related joint pain, and ncRNAs may be involved in regulating the inflammatory response in AS cells.

Overexpression of miR-451 suppressed macrophage migration inhibitory factor (MIF) and levels of inflammatory cytokines (24). Yu et al. (32) found that LOC645166 expression was downregulated in T cells from AS patients, which upregulated NF-κB activation by reducing the recruitment of polyubiquitin chains that block the IKK complex to K63 linkage, making AS patients more sensitive to stimulation by pro-inflammatory cytokines or TLR ligands. Li et al. (33) showed that lncRNA maternally expressed gene 3 (MEG3) plays a partial anti-inflammatory role in AS by targeting miR-146a to regulate the expression of IL-1β, IL-6, and TNF-α. Fang et al. (34) showed that the expression level of hsa_circ_0003307 correlated with the inflammatory response in AS. hsa_circ_0003307 knockdown could reduce the inflammatory response in AS by regulating the PI3K/AKT pathway. Zhang et al. (36) showed that lncRNA H19 in peripheral blood mononuclear cells of AS could form ceRNA with miR22-5p/miR675-5p- vitamin D receptor (VDR) to regulate the IL-17A/IL-23 signaling pathway expression, which has an important role in the pathogenesis of AS. The results of another study showed that hsa_circ_0000652 in peripheral blood mononuclear cells (PBMCs) of AS patients promoted macrophage proliferation and cytokine production and inhibited apoptosis and may act as a pro-inflammatory factor for macrophages and a positive regulator of OX40/OX40L through sponge hsa-miR-1179 (38). Thus, ncRNAs may be involved in regulating the cellular immune inflammatory response in AS, but do not act in the same way.

### 3.2 Osteogenic differentiation of AS cells

The typical pathology of AS involves the progression of inflammation into ossification and ankylosis (51). Pathological osteogenesis can cause progressive ankylosis of the spine and peripheral joints, leading to motor impairment and even permanent loss of mobility, which severely affects patients’ life and work and is the main cause of disability in AS patients (52). Fibroblasts have the potential to differentiate into osteoblasts, as shown by the expression of osteogenic marker genes alkaline phosphatase (ALP) and osteocalcin (OC). However, their activation requires specific cytokine stimulation, the most important one being bone morphogenetic proteins (BMPs) (53). Several studies have shown that ncRNAs regulate the differentiation of fibroblastogenic cells into osteoblasts in AS hip capsule specimens (40, 54). Low expression of miR-150-5p and miR-204-5p in fibroblasts from AS patient ligaments inhibited osteogenesis *via* VDR and Notch2, respectively (25, 26), while high expression of miR-148a-3p exacerbated the osteogenic differentiation of fibroblasts by inhibiting the expression of the downstream target gene Dickkopf homologue 1 (DKK1) and activating the Wnt pathway, leading to increased calcified nodules and mineralization (27). A study of animal experiments showed that miR‐96 was expressed at a high level in proteoglycan-induced AS mice, and overexpression of miR‐96 led to stimulation of osteoblast differentiation and bone formation through activation of the Wnt pathway in AS, which may provide novel aspects for AS treatments in the future (28). A study of osteoblast-specific miR-214 knockout mice (CKO: Ocn-cre; miR-214^fl/fl^ mice) showed that miR-214, the production of which is stimulated by IL-17A in osteoblasts, was an important inhibitor of bone formation in AS. Osteoblast-derived miR-214 stimulated by IL-17A can be transferred into osteoclasts to promote their activity and thus inhibit bone formation (29). Another study revealed that circ_0070562 was significantly upregulated in bone marrow mesenchymal stem cells (BMSCs) from AS patients, which may play an important role in AS-BMSC osteogenesis in combination with miR-424-5p and miR-133b of the TGF-beta pathway (35). Furthermore, in AS hip capsule specimens, circ_0018168 overexpression elevated DKK1 through adsorption of miR-330-3p and inhibited AS fibroblast proliferation and osteogenic differentiation; the results suggested that circ RNA-related ceRNA could play a regulatory role in the osteogenic differentiation of AS cells (39).

### 3.3 AS cell proliferation, apoptosis, and pyroptosis

Excessive proliferation and insufficient apoptosis of many kinds of cells, including T cells, fibroblast-like synoviocytes (FLS), and fibroblasts, lead to the pathogenesis of AS (30, 31, 40, 55). Pyroptosis is an inflammatory type of regulated cell death that occurs following inflammasome activation (56, 57). In AS patients, activated pyroptosis leads to inflammatory responses, which cause various inflammatory diseases (58, 59).

Li et al. (30) found that miR-130a-3p was downregulated in T cells from AS patients, and an miR-130a-3p inhibitor could inhibit T cell proliferation and induce apoptosis by upregulating the downstream target gene HOXB1. Similarly, miR-204 expression was decreased and Gasdermin D (GSDMD) was elevated in the FLS of AS patients. miR-204 mimics inhibited FLS pyroptosis in AS cells by suppressing the expression of GSDMD (31). In addition, ceRNAs are involved in cellular regulation in AS. Li et al. (40) showed that competitive binding of hsa_circ_0056558 and cyclin-dependent kinase 6 (CDK6) to miR-1290 inhibits cell proliferation and differentiation while promoting apoptosis, a process that may be mediated through the PI3K/AKT/NF-κB pathway. Another study showed that lncRNA MALAT1 and GSDMD expression was upregulated in AS chondrocytes, but that of miR-558 was downregulated. Downregulation of lncRNA MALAT1 expression increased miR-558 activity by suppressing GSDMD and inhibited inflammation, apoptosis, and pyroptosis in AS chondrocytes, thus acting as a potential suppressor of AS (37).

## 4 ncRNA applications in AS diagnosis and treatment

The stable and tissue-specific expression of ncRNAs makes them promising diagnostic markers for diseases such as AS; they could help in reflecting the activity of AS, monitoring the effect of treatment, and predicting the occurrence and recurrence rate of AS.

Recent studies have shown that a variety of ncRNAs may have potential diagnostic value and are closely related to AS disease activity. Tan et al. (60) found that miR-146a/miR-125a-5p/miR-125b-5p/miR-499a/miR-155a combination (area under the curve (AUC)=0.824, 95% confidence interval (CI) = 0.727-0.921) had high sensitivity and specificity for the diagnosis of AS. C-reactive protein (CRP) levels were positively correlated with miR-125a-5p (r= 0.438, p = 0.005) and miR-155a (r= 0.414, p = 0.006) expression, suggesting that miR-125a-5p and miR-155a may exacerbate AS-induced inflammation. Another study showed that lnc-ITSN1-2 expression was elevated in patients with AS, and lnc-ITSN1-2 was positively correlated with levels of CRP and interleukin (IL)-1β, Bass Ankylosing Spondylitis Disease Activity Index (BASDAI), and ankylosing spondylitis disease activity with c-reactive protein (ASDASCRP) score (61). Similarly, Tang et al. (62) found that hsa_circRNA_012732 was downregulated during inflammation, and negatively correlated with the BASDAI, high-sensitivity C-reactive protein (hs-CRP), and globulin (GLOB), and positively correlated with lymphocyte count (LY), mean red blood cell volume, and albumin (ALB). ROC curve analysis showed that hsa_circRNA_001544 (95% CI=0.610-0.831, P<0.05) was statistically significant, and its AUC values was 0.720. hsa_circRNA_001544 and hsa_circRNA_012732 have potential to be molecular markers for AS diagnosis and disease activity, respectively. High-throughput sequencing of PBMC samples from five AS patients and healthy controls was performed. NONHSAT118801.2, ENST00000444046, and NONHSAT183847.1 were found to be significantly upregulated in AS patient samples, and the expression of NONHSAT118801.2 and NONHSAT183847.1 was positively correlated with disease severity (63). All these findings suggest that ncRNAs may serve as diagnostic markers for AS.

With respect to therapy, the therapeutic potential of ncRNAs is gradually being revealed as translational medicine research continues to advance. miR-21 can induce the proliferation and differentiation of MSCs to promote bone formation. miR-21-exosome injection may help alleviate spinal osteoporosis in patients with AS, which is characterized by an increase in bone mineral content and bone density and a decrease in osteoclast number (64). However, current studies have mainly focused on animal experiments, and further clinical trials are needed to confirm this in the future.

ncRNAs may predict the occurrence and recurrence rate of AS. Han et al. (65) revealed that lncRNA-adjacent FOXA2 enhancer (lncRNA-NEF) expression was upregulated in synovial fluid samples from AS patients and was associated with ASDAS, BASDAI, erythrocyte sedimentation rate (ESR), and CRP levels (P<0.05). NSAID treatment significantly downregulated lncRNA-NEF expression. Three-year follow-up showed a high relapse rate in patients with high lncRNA-NEF levels (hazard ratio=2.266). These results suggest that lncRNA-NEF upregulation predicts relapse and poor treatment outcome in AS and has great potential as a predictive biomarker for AS relapse. Similarly, Zhong et al. (66) found that LINC00311 was upregulated in AS patients, which positively correlated with disease activity. At 2-year follow-up, patients with high LINC00311 levels had significantly higher rehospitalization rates. Thus, LINC00311 is overexpressed in AS and predicts treatment outcome and recurrence. These results suggest that ncRNA may have an impact on the prognosis of AS.

## 5 Conclusions and future perspectives

**Figure 2:**
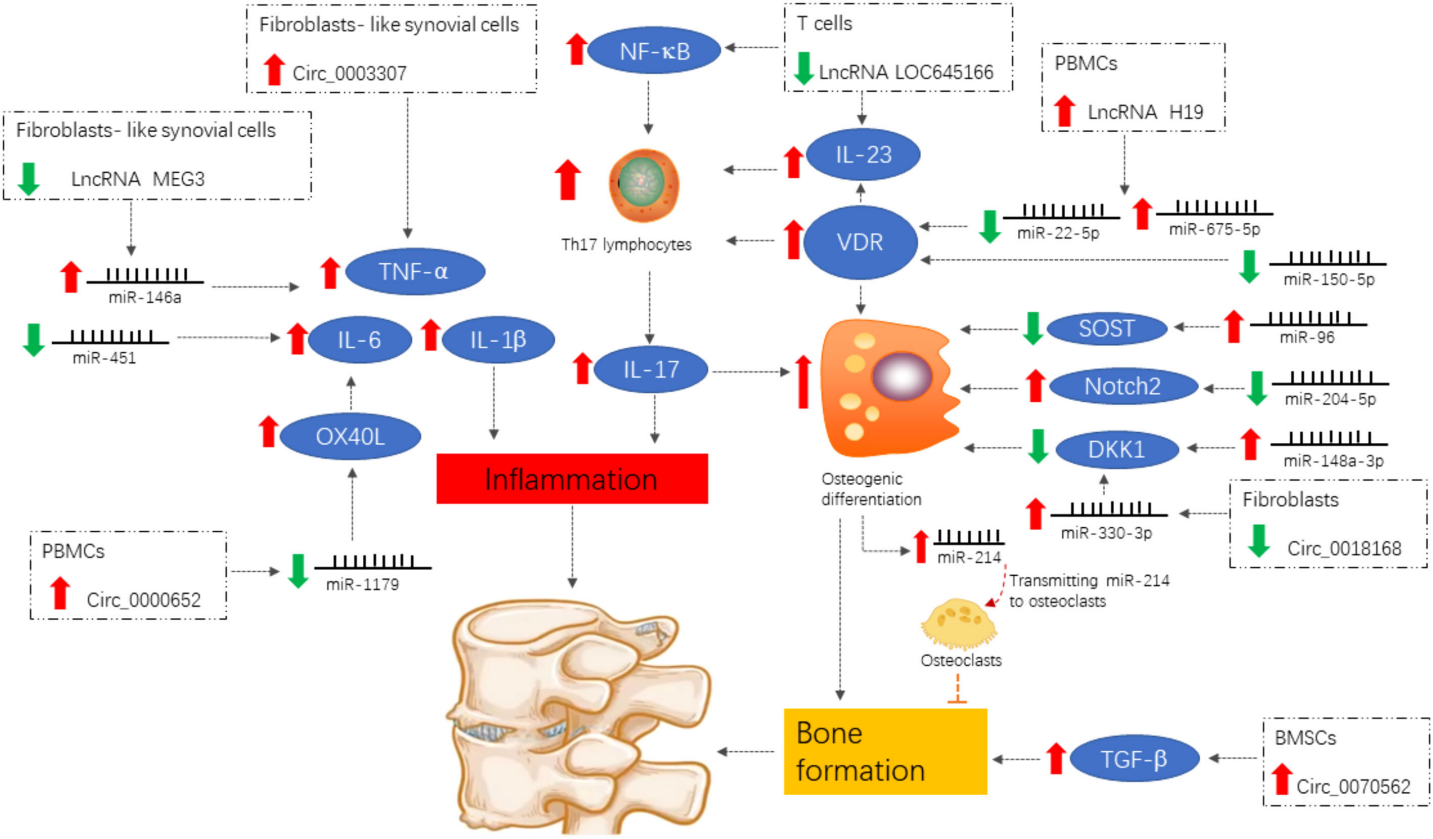
Differentially expressed ncRNAs of functional importance in ankylosing spondylitis. Signals are focused on the induction of the pro-inflammatory cytokines IL-1β, TNF-α, and IL-6 and the enhancement of the IL-23/IL-17 axis, both of which contribute to inflammation and abnormal bone formation.

With the continuous development of molecular biology techniques, our understanding of disease mechanisms has gradually advanced to include genetic and epigenetic processes. As one of the key research areas in epigenetics, ncRNA plays an important regulatory role in diseases including cancers, cardiovascular diseases, and neurological pathologies. ncRNA is an important part of rheumatic disease research to study the pathogenesis and identify new targets for the diagnosis and treatment. Recently, ncRNAs have been studied in association with inflammatory response in AS and bone formation (**Figure 2**); key molecules regulating the developmental process of AS will be identified in the future, providing new entry points for the study of AS etiology and molecular targeting therapy. However, exploring the specific mechanisms of ncRNAs in inflammation and bone formation in AS is challenging due to the wide variety of ncRNAs. Currently, it is difficult to determine which ncRNAs can be targeted for the most effective intervention. In addition, the specific mechanism to intervene in inflammatory responses and bone formation targets *via* ncRNAs is still unclear and needs further investigation.

## Author contributions

YF conceptualized and designed the study. JL supervised the project and contributed to manuscript revision. Both authors contributed to the article and approved the submitted version.
